# Validation of perinatal post-traumatic stress disorder questionnaire for Spanish women during the postpartum period

**DOI:** 10.1038/s41598-021-85144-2

**Published:** 2021-03-10

**Authors:** Antonio Hernández-Martínez, Sergio Martínez-Vázquez, Julian Rodríguez-Almagro, Khalid Saeed Khan, Miguel Delgado-Rodríguez, Juan Miguel Martínez-Galiano

**Affiliations:** 1 Department of Nursing, Faculty of Nursing of Ciudad Real, University of Castilla-La Mancha, C/Cuadras nº 8 Bajo, Ciudad Real, Spain; 2 Department of Nursing, University of Jaen, Jaén, Spain; 3 Department of Preventive Medicine and Public Health, University of Granada, Granada, Spain; 4 Department of Health Sciences, University of Jaen, Jaén, Spain; 5 CIBER de Epidemiología y Salud Pública (CIBERESP), Barcelona, Spain

**Keywords:** Psychiatric disorders, Quality of life

## Abstract

To determine the psychometric properties of the Perinatal Post-Traumatic Stress Disorder (PTSD) Questionnaire (PPQ) in Spanish. A cross-sectional study of 432 Spanish puerperal women was conducted, following ethical approval. The PPQ was administered online through midwives' associations across Spain. The Edinburgh Postnatal Depression Scale was used to diagnose postnatal depression for examining criterion validity. Data were collected on sociodemographic, obstetric, and neonatal variables. An exploratory factorial analysis (EFA) was performed with convergence and criterion validation. Internal consistency was evaluated using Cronbach's α. The EFA identified three components that explained 63.3% of variance. The PPQ's convergence validation associated the risk of PTSD with variables including birth plan, type of birth, hospital length of stay, hospital readmission, admission of the newborn to care unit, skin-to-skin contact, maternal feeding at discharge, maternal perception of partner support, and respect shown by healthcare professionals during childbirth and puerperium. The area under the ROC curve for the risk of postnatal depression (criterion validity) was 0.86 (95% CI 0.82–0.91). Internal consistency with Cronbach's α value was 0.896. The PPQ used when screening for PTSD in postpartum Spanish women showed adequate psychometric properties.

## Introduction

Post-traumatic stress disorder (PTSD), described as the complex somatic, cognitive, affective, and behavioral effects of psychological trauma^1^, is a significant mental health concern for pregnant and postpartum women. PTSD can affect both the mother and the newborn^2^, and it is linked with other mental health issues^3^.

Several tools exist to screen PTSD and, to our knowledge, there is only one tool specific for the perinatal period. The Perinatal PTSD Questionnaire (PPQ) was initially designed by Hynan in 1998^4^. It was composed of 14 items with dichotomous responses. When tested in american population it demonstrated good psychometric properties^5^. In 2006, Callahan et al. changed the dichotomous answers to a Likert scale and re-validated the questionnaire, giving guidance on a cut-off score for clinical^6^. This tool has been examined for its psychometric properties in many languages and cultures including English^7^, Korean^8^, Chinese^9^, and French (in its original dichotomous version)^10^. For a Spanish version, we searched PubMed using kewords, text terms and word variants for the concept ‘Spanish and validation and posttraumatic stress disorder”. There were 50 citation. We found articles describing use of PPQ in the Spanish setting^11, 12^, but there was not a single citation of a validation study in this population.

Spanish is one of the most commonly spoken languages worldwide^13^. In the USA in 2018, it was estimated that 13.5% (41.5 million people) of the population over five years of age speaks Spanish^14^. As no validation study of Spanish PPQ exists and Spanish is an international language, there is a need to examine the performance of Spanish PPQ. This background led us to conduct a study to validate PPQ in Spanish.

## Methodology

An observational cross-sectional validation study was conducted using the modified PPQ questionnaire^6^ in the Spanish language with a sample of postpartum Spanish women who gave birth in Spain with the approval of the Clinical Research Ethics Committee of Universidad de Jaen (reference number TD-VCDEPP-2019/1417-N-19). All participants received written information on the study, including the fact that participation was entirely voluntary with anonymity guaranteed. Before starting the questionnaire, the women had to read an information sheet about the study and its objectives, and check a box in which they showed their consent to participate in the study: that is, they signed an ad hoc digital informed consent.

The study was conducted in accordance with the principles of the Declaration of Helsinki and was conducted in accordance with Strobe cross sectional guidelines.

### Participants

The sample size was estimated in accordance with the criteria for carrying out a factor analysis. These criteria envisage 10 subjects for each item^15^, and therefore we needed a sample of at least 140 participants. This minimum sample size was exceeded. The exclusion criteria for participation in the study were refusal to participate, being less than 18 years of age, and longer than 6 months after childbirth.

### Data collection

An online questionnaire was developed and distributed from November to December 2019 with the collaboration of Spanish midwives' associations. This questionnaire included items collecting sociodemographic and clinical variables, the PPQ and the Edinburgh Postnatal Depression Scale (EPDS).

The following sociodemographic and clinical data were recorded: maternal age, education level, whether or not the pregnancy was desired, live newborn, parity, induction of labor, using natural analgesia, using epidural analgesia, using general anesthesia, type of birth, episiotomy, perineal tear, skin to skin, admission of the newborn to a care unit, degree of partner support, feeling respected by healthcare staff, type of feeding after discharge, surgical intervention, and postnatal hospital readmission. Some of the variables were used to describe the population, and some used in convergence validity.

The second component of the survey consisted of a series of questionnaires. First was the modified PPQ (Appendix S1: Spanish version), a 14-item measure assessing post-traumatic symptoms related to the childbirth experience, including intrusiveness or re-experiencing, avoidance behaviors, and hyperarousal or numbing of responsiveness. The PPQ also contains one item pertaining to feelings of guilt. Response options were modified from the original dichotomous scale to a five-level Likert-type scale (scored 0 to 4). Mothers were instructed to provide responses that reflected their experience during the targeted time frame (1 to 18 months postpartum). The total possible score on the modified PPQ ranged from 0 to 56. In the current investigation, internal consistency was superior to previous investigations using the dichotomous scaling, with an α = 0.90^6^.

The EPDS is a 10-items self-reported scale designed as a specific instrument to detect postnatal depression and has been validated in the Spanish population during pregnancy^16^ and postnatally^17^. With a cut-off point of ≥ 10, the sensitivity was 79% and specificity 95.5%. The positive predictive value was 63.2% and negative predictive value 97.7%^17^. Moreover, it is a simple and widely accepted tool by clinical practitioners^18^. The EPDS was included to establish the criteria validity.

### Data analysis

For sociodemographic and clinical data, the absolute and relative frequencies were used to describe the qualitative variables, and the mean and standard deviation (SD) used to describe the quantitative variables.

First, to determine the validity of the scale used, we analyzed three of the most common validity types: construct validity, convergent validity, and criterion validity.

For construct validity, we opted to carry out an exploratory factor analysis (EFA) to determine the underlying factors through a principal component analysis (PCA). Before carrying out the EFA, we analyzed the Kaiser–Meyer–Olkin (KMO) tests and Bartlett's sphericity tests, to determine whether it was appropriate to apply this analysis. For this to be the case, the KMO should be above 0.6 and as close as possible to 1, and Bartlett's sphericity, which consists of statistical hypothesis testing, should be less than 0.05 to reject the null hypothesis of sphericity and ensure that the factor model is adequate to explain the data. In the EFA, we used Varimax rotation to help clarify the assignation of items to different factors. To determine the number of factors to retain, we used the Kaiser criterion, which is one of the most used criteria. It retains factors with eigenvalues greater than the unit value^19^.

Within the construct validity, we also analyzed convergent validity, in order to establish the relationship between the PPQ and factors which are believed to be associated with PTSD risk, such as type of birth, admission of the newborn to an intensive care unit (NICU), type of feeding, hospital length of stay, among others. Hence, a bivariate analysis was performed using Pearson's chi-squared or Fisher's t-student tests, depending on whether the variable data were qualitative or quantitative. The results were considered statistically significant when *p* < 0.05.

To study criteria validity, the Edinburgh scale was exerted with a ≥ 10 cut-off point. To do this, we carried out a sensitivity and specificity study with an analysis of the area under the received operating characteristic curve (AUC) obtained using Swets' criteria^20^. We also carried out a bivariate analysis between the scores obtained on the PPQ scale and the Edinburgh scale. We again used non-parametrical statistical tests and considered significant associations with *p* < 0.05.

The reliability analysis was done by studying the Cronbach's (α) to evaluate the internal consistency (IC). The IC indicates to what extent the items in the questionnaire are correlated with each other, and how they fit together and measure the same concept. The α is one of the most widely used measures to assess the reliability of a scale^21^. Its values range from 0 to 1. One of the most accepted rules is to consider α > 0.9 as excellent, α > 0.8 as good, α > 0.7 as acceptable, α > 0.6 as questionable, α > 0.5 as poor, and α < 0.5 as unacceptable^22^.

Version 24.0 of the SPSS statistics package was used for analyses.

### Ethical approval

This study was approved by the the approval of the Clinical Research Ethics Committee of Universidad de Jaen (reference number TD-VCDEPP-2019/1417-N-19). Before starting the questionnaire, the participants read a fact sheet about the study, its objectives, etc., and marked a box by which they showed their consent to participate in it, i.e., they signed an online informed consent (ticking the option if they wanted to participate or not doing so when refusing to take part in the study). We followed the protocols established to carry out this type of research with the purpose of publication/disclosure to the scientific community. The study was conducted according to the strobe guidelines set in the Declaration of Helsinki and all procedures involving human subjects were approved by the Ethics Committee. All women involved in this study filled out informed consent and data treatment forms to enter the study, in accordance with the ethical standards of the Ethics Committee. All participants received written information on the study, including the fact that participation was entirely voluntary with anonymity guaranteed.

## Results

### Characteristics of participants

Four hundred thirty-two women agreed to participate in the study and completed the PPQ with a PTSD risk (score ≥ 19) of 11.1% (48). The mean age was 35.4 years (SD = 4.22), and 65.0% (281) were primiparous. Labor was induced in 38.7%, 57.6% (259) had a normal vaginal delivery, and 72.9% (315) needed regional analgesia. Neonatal data showed that 7.9% (34) were admitted to NICU, and 78.0% (337) were exclusively breastfed at the moment of hospital discharge. The remaining descriptive data are detailed in Table 1.

**Table 1:** Characteristics of the sample included in a validation study of the PPQ Spanish questionnaire for PTSD risk.

Variable	Total	PTSD Risk	
	N (%)	Score < 19	Score ≥ 19
**Maternal age**			
Mean (SD)	35.4 (4.22)	5.3 (4.23)	35.8 (4.14)
**Academic Level**			
Primary school	5 (1.2)	5 (100.0)	0 (0.0)
Secondary school	28 (6.5)	24 (85.7)	4 (14.3)
High School	102 (23.6)	94 (92.2)	8 (7.8)
University	297 (68.8)	261 (87.9)	36 (12.1)
**Intended pregnancy**			
No	30 (6.9)	27 (90.0)	3 (10.0)
Yes	402 (93.1)	357 (88.8)	45 (11.2)
**Number of antenatal education sessions**			
No	96 (22.2)	83 (86.5)	13 (13.5)
Less than 5 classes	72 (16.7)	61 (84.7)	11 (15.3)
At least 5 classes	264 (61.1)	240 (90.9)	24 (9.1)
**Birth plan**			
No	227 (52.5)	205 (90.3)	22 (9.7)
Yes, not respected	37 (8.6)	26 (70.3)	11 (29.7)
Yes, and respected	168 (38.9)	153 (91.1)	15 (8.9)
**Twin pregnancy**			
No	423 (97.9)	376 (88.9)	147 (11.1)
Yes	9 (2.1)	8 (88.9)	1 (11.1)
**Gestational age**			
Term	406 (94.0)	364 (89.7)	42 (10.3)
Preterm (32–37 w)	21 (4.9)	18 (85.7)	3 (14.3)
Very preterm (28–32 w)	2 (0.5)	1 (50.0)	1 (50.0)
Extreme preterm (< 28 s)	3 (0.7)	1 (33.3)	2 (66.7)
**Live newborn**			
No	2 (0.5)	1 (50.0)	1 (50.0)
Yes	432 (99.5)	383 (89.1)	47 (110.9)
**Parity**			
Primiparous	281 (65.0)	246 (87.5)	35 (12.5)
Multiparous	151 (35.0)	138 (91.4)	13 (8.6)
**Induction of labor**			
No	265 (61.3)	241 (90.9)	24 (1)
Yes	167 (38.7)	143 (85.6)	24 (14.4)
**Natural analgesia**			
No	349 (80.8)	307 (88.0)	42 (12.0)
Yes	83 (19.2)	77 (92.8)	6 (7.2)
**Regional analgesia**			
No	117 (27.1)	104 (88.9)	13 (11.1)
Yes	315 (72.9)	280 (88.9)	35 (11.1)
**General anesthesia**			
No	421 (97.5)	376 (89.3)	45 (10.7)
Yes	11 (2.5)	8 (72.7)	3 (27.3)
**Type of birth**			
Normal birth	249 (57.6)	232 (93.8)	17 (6.8)
Instrumental	91 (21.1)	81 (89.0)	10 (11.0)
Elective CS	27 (6.3)	24 (88.9)	3 (11.1)
Emergency CS	65 (15.0)	47 (72.3)	18 (27.7)
**Episiotomy**			
No	308 (71.3)	270 (87.7)	38 (12.3)
Yes	124 (28.3)	114 (91.9)	10 (8.1)
**Perineal Tear**			
No	258 (59.7)	224 (86.8)	34 (13.2)
Minor	160 (37.0)	104 (93.1)	11 (6.9)
Severe	14 (3.2)	11 (78.6)	3 (6.9)
**Skin to skin**			
No	94 (21.8)	72 (76.6)	22 (23.4)
Yes	338 (78.2)	312 (92.3)	26 (7.7)
**Neonatal Admission**			
No	372 (86.1)	342 (91.9)	30 (8.1)
Intermediate care unit	26 (6.0)	16 (61.5)	10 (38.5)
NICU	34 (7.9)	26 (7.5)	8 (23.5)
**Hospital length of stay**			
1 day	31 (7.2)	28 (90.3)	3 (9.7)
2 day	215 (49.8)	203 (94.4)	12 (5.6)
3 day	120 (27.8)	104 (86.7)	16 (13.3)
4 days or more	66 (15.3)	49 (74.2)	17 (25.8)
**Partner Support**			
None	9 (2.1)	4 (44.4)	5 (55.6)
Little	15 (3.5)	10 (66.7)	5 (33.3)
Some	32 (7.4)	26 (81.3)	6 (18.8)
Quite	94 (21.8)	84 (81.3)	10 (10.6)
A lot	282 (65.3)	260 (92.2)	22 (7.8)
**Feeling respected by healthcare staff**			
None	19 (4.4)	7 (36.8)	12 (63.2)
Little	24 (5.6)	15 (62.5)	9 (37.5)
Some	66 (15.3)	58 (87.9)	8 (12.1)
Quite	153 (35.4)	137 (89.5)	16 (10.5)
A lot	170 (39.4)	167 (88.2)	3 (1.8)
**Feeding at discharge**			
Maternal	337 (78.0)	309 (91.7)	28 (8.3)
Mixed	79 (18.3)	63 (79.7)	16 (20.3)
Artificial	16 (3.7)	12 (75.0)	4 (25.0)
**Postnatal surgical intervention**			
No	415 (96.1)	374 (90.1)	41 (9.9)
Yes	17 (3.9)	10 (58.8)	7 (41.2)
**Hospital readmission**			
No	417 (96.5)	371 (89.0)	46 (11.0)
Yes	15 (3.5)	13 (86.7)	2 (13.3)

CS, Cesarean section; NICU, Neonatal Intensive Care Unit.

### Psychometric properties

#### Factor construct validity

The KMO test gave a value of 0.902, and Bartlett's sphericity test was < 0.01. Therefore, we proceeded to carry out the EFA. Three components explained 63.3% of the variance. The first component, "Arousal," consisted of items 7, 8, 9, 10, 11, 12, and 13 and accounted for 43.0% of variance. The second component, "Avoidance," consisted of items 2, 4, 5, 6, and 14 explained 13.8% of variance, while the third component "Intrusion" was formed by two items, 1 and 3, accounted for 6.3% of total variance. Furthermore, all the anti-image diagonal correlations showed figures higher than 0.86. Table 2 presents the scale items together with their respective factor weights.

**Table 2:** Rotated component matrix.

Item	Components		
	1 Arousal	2 Avoidance	3 Intrusion
Q1	0.188	0.184	**0.822**
Q2	0.162	**0.706**	0.341
Q3	0.087	0.331	**0.704**
Q4	0.218	**0.768**	0.234
Q5	0.163	**0.830**	0.084
Q6	0.142	**0.681**	0.121
Q7	**0.764**	0.122	0.139
Q8	**0.797**	0.206	0.152
Q9	**0.735**	0.283	0.003
Q10	**0.721**	0.097	0.226
Q11	**0.831**	0.38	-0.031
Q12	**0.660**	0.182	0.284
Q13	**0.779**	0.179	0.034
Q14	0.486	**0.531**	0.107
Distribution of the components according to validation version			
Spanish	7, 8, 9, 10, 11, 12, 13	2, 4, 5, 6, 14	1, 3
English	7, 8, 10, 12	2, 4, 5, 14	1, 3, 13
Korean	7,8, 9, 10, 11, 12, 13,	4, 5, 6	1, 2, 3, 14
Chinese	7, 8, 9, 10, 11, 12, 13	4, 5, 14	1, 2, 3

#### Convergent validity

Next, the convergent validity was analyzed using bivariate analysis of the scores from the PPQ questionnaire and various sociodemographic and clinical factors. A statistically significant relationship was observed between PTSD risk with the following variables: Birth plan, type of birth, hospital length of stay, hospital readmission, skin to skin, admission of the newborn to NICU, degree of partner support, feeling respected by healthcare staff, and type of feeding on discharge.

#### Criterion validity

**Figure 1:**
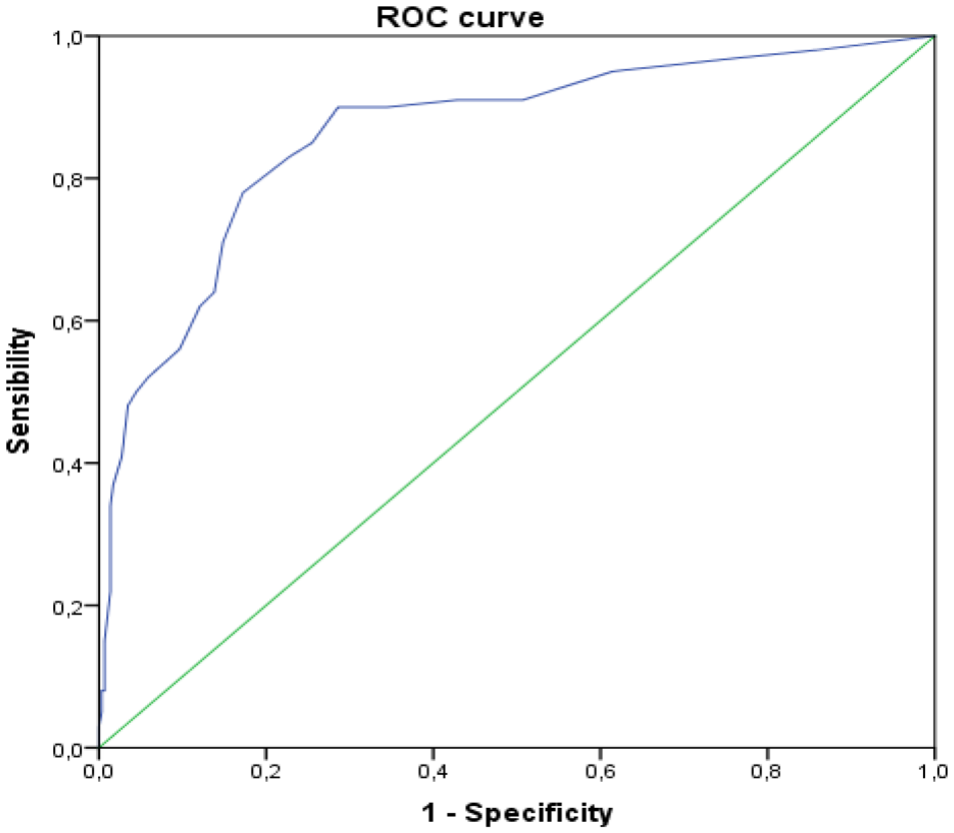
ROC curve. Predictive capacity of the score in the PPQ for PD risk using the Edinburgh questionnaire.

Using the EPDS as a comparative instrument, it was found that the PPQ, translated and transculturally adapted into Spanish, presented an AUC of 0.86 (95% CI 0.82–0.91), with a good capacity to classify the subjects according to Swets' criteria. The ROC curve can be seen in Fig. 1. The bivariate analysis between the scores of the PPQ and Edinburgh scales shows a significant positive relationship (r = 0.69, *p* < 0.001).

#### Internal consistency

To evaluate internal consistency, the α of the total of the questionnaire was used, as well as that of each of the dimensions found with the EFA. For the total scale, α was 0.896. All the alfa values scored higher than 0.880 when removing an item, and the general α did not increase by more than 0.01; therefore, we decided to keep them. The α values for each factor are shown in Table 3.

**Table 3:** Internal consistency of the PPQ.

Variable	Cronbach's alpha (α)
**Total**	0.896
**When removing the item:**	
(1) Did you have bad dreams of giving birth or of your baby's hospital stay?	0.894
(2) Did you have upsetting memories of giving birth or of your baby's hospital stay?	0.891
(3) Did you have any sudden feelings as though your baby's birth was happening again?	0.895
(4) Did you try to avoid thinking about childbirth or your baby's hospital stay?	0.889
(5) Did you avoid doing things that might bring up feelings you had about childbirth or your baby's hospital stay (e.g., not watching a TV show about babies)?	0.892
(6) Were you unable to remember parts of your baby's hospital stay?	0.894
(7) Did you lose interest in doing things you usually do (e.g., did you lose interest in your work or family)?	0.886
(8) Did you feel alone and removed from other people (e.g., did you feel like no one understood you)?	0.881
(9) Did it become more difficult for you to feel tenderness or love with others?	0.885
(10) Did you have unusual difficulty falling asleep or staying asleep?	0.887
(11) Were you more irritable or angry with others than usual?	0.885
(12) Did you have greater difficulties concentrating than before you gave birth?	0.886
(13) Did you feel more jumpy (e.g., did you feel more sensitive to noise, or more easily startled)?	0.885
(14) Did you feel more guilt about the childbirth than you felt you should have felt?	0.886

## Discussion

Our analyses demonstrate the internal consistency, and construct and criterion validity of the Spanish PPQ. This allows for confidence in the use of the PPQ tool in a Spanish setting, something that had not been asured prior to our study.

Another important aspect to consider is the detected prevalence of PTSD risk, which stood at 11.1% in our sample. Apparently, it can be high, however, the Prevalence of PTSD is variable depending on the established cut-off point and the study population type. In 2017, a systematic review and meta-analysis of PTSD reported prevalence rates of 4.0% (95% CI 2.77–5.71) in the general population, with 18.5% (95% CI 10.6–30.38) of women at risk^23^. In addition, it should be clarified that the PPQ tool has a screening and not a diagnostic purpose, therefore it is normal that it presents a higher prevalence than the diagnosed cases.

With regard to factor construct validity the values obtained in KMO tests and Bartlett's sphericity test were adequate; thus, we conducted the EFA. Three components accounted for 63.3% of variance. The English, Korean, and Chinese versions explained 65%, 67%, and 51%, respectively, of variance^7–9^. Regarding components distribution, none of them match among the published versions, as can be seen in Table 2. Moreover, the Korean, Chinese, and Spanish versions coincide in the first component items.

The questionnaire presents an adequate convergent validity as is associated with variables linked previously with PTSD risk, such as type of birth^11, 12, 23–30^, prematurity^5, 31^, neonatal admission to care unit^25^, skin-to-skin^11^, and type of feeding^23^. Furthermore, other associations with PTSD risk were observed, including the degree of partner support and feeling respected by healthcare staff. We also used the same cut-off point (≥ 19) used by Callahan et al.^6^ to consider the risk of PTSD, being the same as that considered by the authors for clinical application; thus bringing the validation closer to its true clinical setting application.

The criterion validity was then evaluated using the EPDS. I opted for this tool because other authors have observed a strong correlation between PTSD and postpartum depression (33), it is a very well-known instrument used by professionals in clinical practice^18^. Specifically, we used EPD scores of ≥ 10 for determining the predictive capacity, finding almost excellent ROC AUC values.

Finally, internal consistency was evaluated and we found values very close to those found in the English version (Cronbach's α = 0.90)^7^ and for the Korean version (Cronbach's α = 0.91)^8^. The lowest internal consistency values were found in the Chinese version (Cronbach's α = 0.837)^9^, and especially in the French version (Cronbach's α = 0.77)^10^. This large difference could be explained because all the versions except the French used Likert-type scale while the French version used dichotomous responses.

With the validation of this questionnaire, practitioners can count on a new tool to identify those women who are at risk of developing PTSD after delivery. The tool is simple and easy to apply, in such a way that it could be included as another assessment tool during the postpartum period, just as the EPDS is used almost systematically for PD screening^18^. Health professionals with this type of tool can direct efforts towards the early detection and prevention of the consequences of a prevalent problem with an increasing trend and that has important consequences for the health of women and their offspring^32, 33^.

The validation of this instrument has a special relevance in the field of PTSD research, since to date there is no specific instrument for assessing the risk of perinatal PTSD in the Spanish-speaking population. Validations, as recommended by scientific societies^34^, they are essential so that researchers can use the assessment instruments in future research and can obtain valid results, establish comparisons, and measure the impact on women's health.

The strengths of our study include the opportunity to evaluate the Spanish PPQ across a diverse sample of a sociodemographically and clinically varied group of puerperal women. We had ample sample size for our evaluation with precisions. We also decided to only include women who had given birth 6 months ago to reduce memory bias as much as possible. There are various limitations of our study. Once the women who declined to take part in the study had been considered, there was no reason to believe there had been any selection bias as the number of non-participants was small, and the sample was consecutively selected. Regarding information bias, using an online questionnaire to collect data could be a limitation due to the lack of access that some women may experience, however, Callahan et al. already used this system for validation previously^6^.

## Conclusions

In conclusion, the validation of the Spanihs PTSD Questionnaire, PPQ in postpartum Spanish women, showed adequate psychometric characteristics, which makes it appropriate for clinical practice in Spanish setting.

## Supplementary Information

Supplementary Information

Supplementary Information

## Data Availability

The data sets generated and/or analysed during the current study are available from the corresponding author o reasonable request.
