# Supplementary material for: Validation of perinatal post-traumatic stress disorder questionnaire for Spanish women during the postpartum period
Source: Sci Rep. 2021 Mar 10;11:5567. doi: 10.1038/s41598-021-85144-2 (PMC7946897; doi:10.1038/s41598-021-85144-2)
Supplement: Supplementary file 1 — Supplementary Information [file 41598_2021_85144_MOESM1_ESM.docx]

**Validation of Perinatal Post-Traumatic Stress Disorder Questionnaire for Spanish Women during the Postpartum Period**

**Appendix A: Spanish Version of PPQ (Versión Española PPQ).**

¿Valore con qué frecuencia vive estas situaciones?

|  | Nada (0) | Una o dos veces (1) | A menudo, pero menos de un mes (2) | A menudo, pero más de un mes (3) |
| --- | --- | --- | --- | --- |
| ¿He tenido sueños o pesadillas relacionados con el nacimiento de mi bebé? |  |  |  |  |
| ¿Me trae malos recuerdos pensar en mi parto o en la estancia de mi bebé en el hospital? |  |  |  |  |
| ¿He tenido la sensación repentina de que mi parto volvía a ocurrir (flashback)? |  |  |  |  |
| ¿Intento o he intentado evitar pensar en lo relacionado con el nacimiento de mi hijo/a? |  |  |  |  |
| ¿Evito hacer cosas que me recuerden el parto o la estancia en el hospital? |  |  |  |  |
| ¿Tengo lagunas o me cuesta recordar momentos de mi estancia hospitalaria? |  |  |  |  |
| ¿Noto menos interés en cosas con las que antes sí disfrutaba? |  |  |  |  |
| ¿Me siento sola o distanciada de los demás? |  |  |  |  |
| ¿Me ha resultado difícil sentir cariño o amor por los que me rodean? |  |  |  |  |
| ¿Estoy teniendo dificultades para dormir? |  |  |  |  |
| ¿Me he sentido más enfadada con los demás que anteriormente? |  |  |  |  |
| ¿Tengo dificultades para concentrarme a raíz del parto? |  |  |  |  |
| ¿Me noto más irritable? (Como si por ejemplo el ruido me molestara más de lo habitual). |  |  |  |  |
| ¿Me siento más culpable respecto a las circunstancias que rodearon el nacimiento de mi hijo de lo que me parecería normal? |  |  |  |  |
