# Supplementary material for: Validation of perinatal post-traumatic stress disorder questionnaire for Spanish women during the postpartum period
Source: Sci Rep. 2021 Mar 10;11:5567. doi: 10.1038/s41598-021-85144-2 (PMC7946897; doi:10.1038/s41598-021-85144-2)
Supplement: Supplementary file 2 — Supplementary Information [file 41598_2021_85144_MOESM2_ESM.docx]

**Validation of Perinatal Post-Traumatic Stress Disorder Questionnaire for Spanish**

**Women during the Postpartum Period**

**Antonio Hernández-Martínez ^1^, Sergio Martinez-Vazquez^2^ , Julian Rodríguez-Almagro ^1*^, Khalid Saeed Khan^3^, Miguel Delgado-Rodríguez ^4,5^, and Juan Miguel Martínez-Galiano^,2,5^**

1. Department of Nursing. Faculty of Nursing of Ciudad Real. University of Castilla-La Mancha, Ciudad Real, Spain
2. Department of Nursing of University of Jaen, Jaén, Spain
3. Department of Preventive Medicine and Public Health, University of Granada, Granada, Spain.
4. Department of Health Sciences of University of Jaen, Spain.
5. CIBER de Epidemiología y Salud Pública (CIBERESP), Spain.

***Corresponding Author:** julianj.rodriguez@uclm.es; **Tel.:** +34676683843; **E-mail address:** julianj.rodriguez@uclm.es; **Mailing address:** C/Cuadras nº 8 Bajo; Ciudad Real, Spain

**Author contributions statement**

Antonio Hernández-Martínez: Visualizaton, Conceptualization, Methodology, Software.

Sergio Martínez-Vazquez: Investigation, Reviewing.

Julián Rodríguez-Almagro: Data curation, Writing- Original draft preparation, Reviewing and Editing, Supervision .

Khalid Saeed Khan : Visualization, Investigation.

Miguel Delgado-Rodríguez: Visualization, Investigation

Juan Miguel Martínez-Galiano: Supervision, Writing- Reviewing and Editing.

**Competing interests statement**

The authors(s) declare no competing interests. No conflict of interest has been declared by the author(s). This research received no specific grant from any funding agency in the public, commercial, or not-for-profit sectors.

**Data availability statement**

The data sets generated and/or analysed during the current study are available from the corresponding author o reasonable request.

**Ethical approval**

This study was approved by the the approval of the Clinical Research Ethics Committee of Universidad de Jaen

(reference number TD-VCDEPP-2019/1417-N-19). Before starting the questionnaire, the participants read a

fact sheet about the study, its objectives, etc., and marked a box by which they showed their consent to participate in

it, i.e., they signed an online informed consent (ticking the option if they wanted to participate or not doing so when

refusing to take part in the study). we followed the protocols established to carry out this type of research with the

purpose of publication/disclosure to the scientific community. The study was conducted according to the strobe

guidelines set in the Declaration of Helsinki and all procedures involving human subjects were approved by the

Ethics Committee. All women involved in this study filled out informed consent and data treatment forms to enter

the study, in accordance with the ethical standards of the Ethics Committee.

All participants received written information on the study, including the fact that participation was entirely

voluntary with anonymity guaranteed.

**Acknowledgement**

Professor Khan is Distinguished Investigator at University of Granada funded by the Beatriz Galindo (senior modality) program of the Spanish Ministry of Education.
